# Use of a Javid™ shunt in the management of axillary artery injury as a complication of fracture of the surgical neck of the humerus: a case report

**DOI:** 10.1186/1752-1947-2-259

**Published:** 2008-08-05

**Authors:** Stuart A Suttie, Reza Mofidi, Alison Howd, Gareth D Griffiths

**Affiliations:** 1 Department of Surgery and Molecular Oncology, Ninewells Hospital and Medical School, Dundee DD1 9SY, UK; 2 Department of Vascular Surgery, Ninewells Hospital, Dundee DD1 9SY, UK

## Abstract

**Introduction:**

Axillary artery injury is a rare but severe complication of fractures of the surgical neck of the humerus.

**Case presentation:**

We present a case of axillary artery pseudoaneurysm secondary to such a fracture, in a 82-year-old white woman, presenting 10 weeks after the initial injury, successfully treated with subclavian to brachial reversed vein bypass together with simultaneous open reduction and internal fixation of the fracture. We discuss the use of a Javid™ shunt during combined upper limb revascularisation and open reduction and internal fixation of the fractured humerus.

**Conclusion:**

This case highlights the usefulness of a Javid™ shunt, over other forms of vascular shunts, in prompt restoration of blood flow to effect limb salvage. It can be considered as a temporary measure whilst awaiting definitive revascularisation which can be performed following fracture fixation.

## Introduction

Proximal humeral fractures are a common injury with an incidence of approximately 5% of all fractures, with the majority being secondary to blunt trauma in an elderly population [1]. Despite the close proximity of the axillary artery and the surgical neck of humerus, injury to this artery is a rare complication of proximal humeral fractures. It is, however, associated with significant risks to both function and viability of the affected upper limb.

Upper limb ischaemia secondary to such a cause requires prompt intervention to restore blood flow and subsequently treat the primary cause. Earlier reports have documented success in similar settings, using modified equipment not necessarily designed for use as an intravascular shunt [2,3].

We present a case of delayed presentation of axillary artery pseudoaneurysm following proximal humeral fracture and discuss the use of a Javid™ carotid shunt (Bard carotid shunt, 17F tapered to 10F; Bard^® ^Javid™ Carotid Shunts, Bard Ltd., Forest House, Brighton Rd., Crawley, West Sussex, UK) in maintaining vascular perfusion during open reduction and internal fixation of the fracture.

## Case presentation

**Figure 1:**
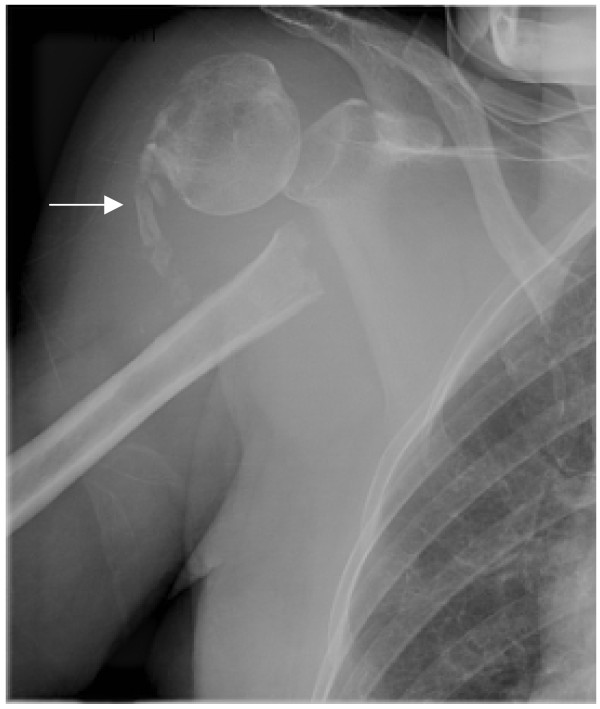
Anteroposterior view of right shoulder 10 weeks after the primary injury, revealing malalignment of fracture ends and attempts at formation of primary callus (arrow).

An 82-year-old, white woman with a history of alcohol abuse, presented to the accident and emergency department with a 4-hour history of an acutely ischaemic right upper limb with motor and sensory deficit. A hard tender, pulsatile mass was palpable in the right subclavian area with significant bruising; there was a palpable right subclavian pulse with no pulses distal to this. X-ray revealed a fracture of the surgical neck of the right humerus with the humeral head abducted and externally rotated, while the humeral shaft was displaced medially (Fig. 1).

Ten weeks previously, she had presented with a fracture of the surgical neck of the right humerus following a fall whilst under the influence of alcohol. On that occasion, sensory and motor function of the limb had been recorded to be fully intact by the medical staff in Accident and Emergency and there had been a full complement of pulses. Given she had no neuro-vascular deficit in the affected limb, the vascular surgeons were not involved initially. Under guidance of the orthopaedic surgeons, she had been treated conservatively with a collar and cuff due to her age and history of current alcohol abuse. She was to have been followed up fortnightly in the orthopaedic fracture clinic – but failed to attend after her second visit. She had no neuro-vascular deficit on follow-up. She denied any further falls or trauma to the right upper limb.

**Figure 2:**
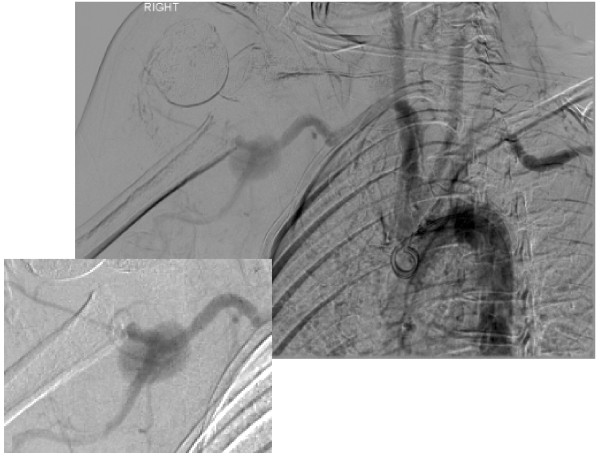
Catheter angiogram depicting pseudoaneurysm formation of third part of axillary artery with complete occlusion of the distal right brachial artery.

The acute nature of the current presentation together with neurological compromise prompted classification as category-II acute limb ischaemia (Society for Vascular Surgery/International Society for Cardiovascular Surgery classification) [4] and urgent angiography was performed with a view to revascularisation. This revealed a pseudoaneurysm of the third part of the right axillary artery with complete occlusion of the right brachial artery distal to this (Fig. 2).

**Figure 3:**
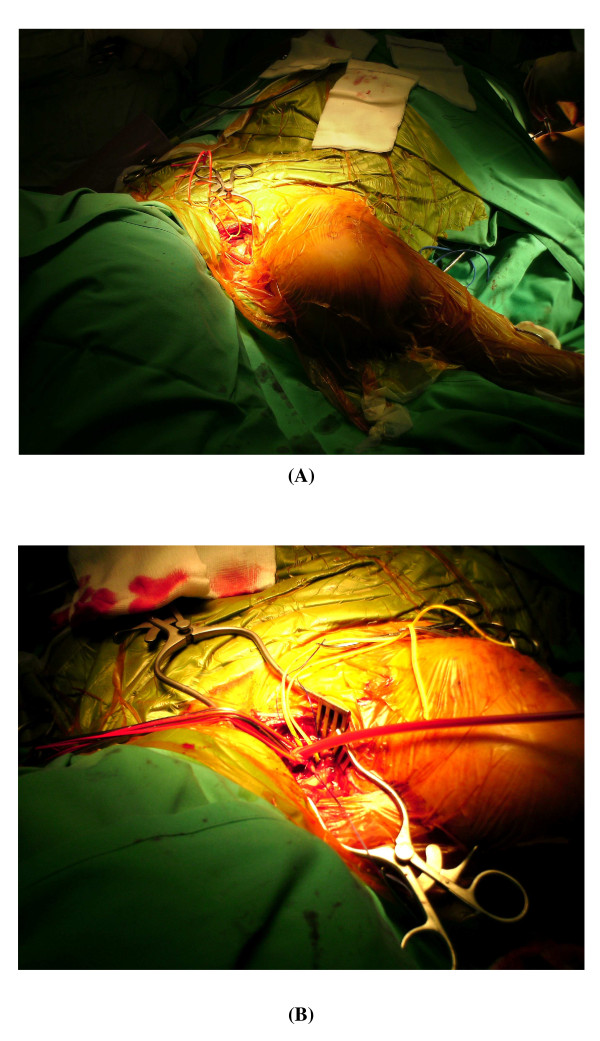
**Supraclavicular exposure of the subclavian artery**. (A) The phrenic nerve is retracted before the division of the scalenus anterior muscle. (B) The subclavian artery is exposed and ligated distally, with blood flow to the right arm being maintained with the aid of a Javid shunt during open reduction and internal fixation of the fracture.

Operative treatment was undertaken with initial exposure and control of the subclavian artery above the clavicle (Fig. 3A). Simultaneous exposure of the brachial artery in the antecubital fossa was performed and a size 3 Fogarty embolectomy catheter passed distally down the brachial artery. Both radial and ulnar arteries were found to contain thrombus which was cleared with good back flow. The proximal brachial and distal subclavian arteries were ligated in continuity. Two interconnected Javid™ shunts were inserted to carry blood flow from the subclavian to the brachial artery in order to maintain perfusion (Fig. 3B) during open reduction and internal fixation of the fractured humerus, after which a subclavian to brachial bypass was performed using reversed long saphenous vein. The fracture was temporarily stabilised using external splints to immobilize the limb whilst securing vascular continuity.

Postoperatively, the patient had strong radial and ulnar pulses with complete resolution of her motor and sensory dysfunction within 72 hours. Her postoperative course was uncomplicated and she was discharged on the 10th postoperative day. Early postoperative duplex scan performed at 6 weeks revealed satisfactory function of the vein graft.

## Discussion

Despite the fact that a significant proportion of fractures of the surgical neck of the humerus are displaced, axillary artery injuries secondary to these fractures are rare [1,4-7]. The majority affect the third part of the artery, due to its position of relative immobility, being tethered by the subscapular and thoracromial arteries [1,8]. Most of these injuries lead to thrombosis of the axillary artery and acute lower limb ischaemia [4,5,9]. Pseudoaneurysm formation of the axillary artery is rare following blunt and penetrating trauma to the shoulder, often presenting late as a pulsatile mass rather than acute limb ischaemia [1,6,7,10].

Endovascular treatment with a covered stent graft has been reported previously and is the treatment of choice in patients with pseudoaneurysm of the axillary artery without upper limb ischaemia [7,11]. Due to the presence of propagating thrombus and displaced fracture requiring open reduction and internal fixation, endovascular treatment was not an option in this patient. Following proximal and distal arterial control and thrombectomy, the limb was revascularised temporarily using a Javid™ shunt, which allowed safe internal fixation of the fracture before bypass grafting. The insertion of the Javid™ shunt served to confirm the viability of the limb and adequacy of distal thrombo-embolectomy. The use of temporary shunting of peripheral vasculature in order to maintain distal vascular perfusion is rarely employed in civilian surgical practice [2,3], however, it has been gaining popularity in the management of military trauma [12-14]. Recent reports from Belfast, whereupon the use of intraluminal shunts has been advocated for the early restoration of blood flow following complex lower limb vascular injuries, have shown significant benefits in averting the incidence of fasciotomy, contractures, ischaemic nerve palsy and amputations [15]. This Belfast approach of early shunting allows for a disciplined surgical approach with adequate time for wound debridement, safe fracture fixation and optimal vascular reconstruction. Reports from Operation Iraqi Freedom suggest that vascular shunts can be used safely to bypass complex vascular injuries encountered in forward surgical units, in order to allow transfer of injured patients for definitive vascular assessment and reconstruction [12,14]. The use of vascular shunts in these circumstances was associated with very low limb amputation rates [14], even in patients in whom the shunt had thrombosed in transit [12].

The Javid™ shunt has the advantage over other types of non-vascular shunt employed [2,3], in that it is specifically designed for use as a carotid artery shunt. It is manufactured out of soft, kink free material, which is tapered towards the ends which are bulbous in nature. This allows the shunt to be clamped in place around the artery, thereby providing stability whilst surgery continues. It was felt that the Javid™ shunt was superior to the Pruitt-Inahara^® ^carotid shunt (an H-shaped carotid shunt, held in place using inflatable balloons) for this patient due to its ease of use, lack of extra lumens (which would easily be caught and cause the shunt to be dislodged), ability to interconnect two shunts and its specially designed clamps to hold the shunt in situ during extensive and vigorous mobilisation of the fractured bone during reduction and fixation. Although these shunt clamps may cause more damage to the arterial lumen than the balloon of the Pruitt-Inahara^® ^shunt, this damaged segment of the injured artery would in turn be ligated and bypassed.

## Conclusion

This case highlights the usefulness of a Javid™ shunt, over other forms of shunt, in prompt restoration of blood flow to effect limb salvage. It can be considered as a temporary measure whilst awaiting definitive revascularisation which can be performed following fracture fixation.

## Competing interests

The authors declare that they have no competing interests.

## Authors' contributions

SAS was first assistant (subclavian exposure), carried out the literature review and constructed the manuscript. RM was first assistant (brachial exposure), photographer, carried out the literature review and drafted and editing the manuscript. AH was primary surgeon (brachial exposure), constructed the idea behind the case report, was senior editor of the manuscript (critical revisions) and gave final approval. GDG was primary surgeon (subclavian exposure), constructed the idea behind the case report, was senior editor of the manuscript (critical revisions) and gave final approval.

## Consent

Written informed consent was obtained retrospectively from the patient for publication of this case report and accompanying images. A copy of the written consent is available for review by the Editor-in-Chief of this journal.
